# Divergent motor projections from the pedunculopontine nucleus are differentially regulated in Parkinsonism

**DOI:** 10.1007/s00429-013-0579-6

**Published:** 2013-05-26

**Authors:** Cristina Martinez-Gonzalez, Judith van Andel, J. Paul Bolam, Juan Mena-Segovia

**Affiliations:** MRC Anatomical Neuropharmacology Unit, Department of Pharmacology, University of Oxford, Mansfield Road, Oxford, OX1 3TH UK

**Keywords:** Brainstem, Locomotion, Subthalamic nucleus, Reticulospinal neurons, Parkinson’s disease, Immediate early genes

## Abstract

The pedunculopontine nucleus (PPN) is composed of neurons with different connectivity patterns that express different neurochemical markers, display distinct firing characteristics and are topographically organized in functional domains across its rostro-caudal axis. Previous reports have shown that the caudal region of the PPN is interconnected with motor regions of both the basal ganglia and brainstem/medulla. The co-distribution of ascending and descending motor outputs raises the question as to whether the PPN provides a coordinated or differential modulation of its targets in the basal ganglia and the medulla. To address this, we retrogradely labeled neurons in the two main PPN pathways involved in motor control and determined whether they project to one or both structures, their neurochemical phenotype, and their activity in normal and dopamine depleted rats, as indicated by Egr-1 expression. We show that ascending and descending motor pathways from the PPN arise largely from separate neurons that intermingle in the same region of the PPN, but have a distinct neurochemical composition and are differentially regulated in the Parkinsonian state. Thus, neurons projecting to the subthalamic nucleus consist of cholinergic, calbindin- and calretinin-expressing neurons, and Egr-1 is upregulated following a 6-hydroxydopamine lesion. In contrast, a larger proportion of neurons projecting to the gigantocellular nucleus are cholinergic, none express calbindin and the expression of Egr-1 is not changed by the dopamine lesion. Our results suggest that ascending and descending motor connections of the PPN are largely mediated by different sets of neurons and there are cell type-specific changes in Parkinsonian rats.

## Introduction

The pedunculopontine nucleus (PPN) is situated in the brainstem and was originally proposed to be an interface between the basal ganglia and the spinal cord (Garcia-Rill 1986). Considered as a part of the mesencephalic locomotor region (MLR), early experiments in decorticated rats showed that electrical stimulation of this region and its surroundings induced a motor response (Skinner et al. 1990), thus providing evidence of its descending control of locomotion. Tracer studies have shown that the PPN has descending projections that mainly innervate targets in the lower brainstem and medulla, including the nucleus reticularis pontis oralis, the gigantocellular nucleus (GiN) and the medioventral medulla, and then innervate some regions of the spinal cord (Mitani et al. 1988; Semba et al. 1990; Rye et al. 1988; Grofova and Keane 1991; Nakamura et al. 1989; Scarnati et al. 2011). These projections have been implicated in the control of gait and posture.

In addition to the descending projections, neurons of the PPN provide an extensive ascending axonal innervation of several neuronal systems across the midbrain and forebrain, including the basal ganglia. Among these, the relationship between the subthalamic nucleus and the PPN (Nomura et al. 1980; Edley and Graybiel 1983; Hammond et al. 1983) has recently attracted the attention in clinical neuroscience given the proposed roles of both structures in the pathophysiology of Parkinson’s disease (PD) and their current use as therapeutic targets for deep brain stimulation (DBS). The use of DBS in the PPN has suggested its involvement in the control of gait (Kringelbach et al. 2007; Thevathasan et al. 2012; Wilcox et al. 2011; Moro et al. 2010), even though this therapy has yielded ambiguous results (Hamani et al. 2011).

The PPN is composed of three main phenotypes of neurons, cholinergic, GABAergic and glutamatergic (Mena-Segovia et al. 2009; Wang and Morales 2009), but further subgroups have been defined on the basis of the expression of neurochemical markers (Martinez-Gonzalez et al. 2012) and electrophysiological properties (Ros et al. 2010). Along with the neuronal heterogeneity, important topographical differences define the connectivity of PPN neurons and their functionality (Martinez-Gonzalez et al. 2011). Neurons innervating motor structures are predominantly situated in the caudal part of the PPN, which raises the possibility that basal ganglia and medulla receive the same signal from PPN neurons. Alternatively, ascending and descending motor projections may be segregated, arising from different populations of PPN neurons. These possibilities entail two different scenarios, the first one consisting of a coordinated activation of upstream and downstream targets, and the second supports the idea of a differential motor output where PPN afferents, as well as local circuitry, play an important part in determining which pathway is activated.

Using anatomical methods, we characterized the connectivity of PPN neurons with two key motor structures from the ascending and descending PPN motor pathways (STN and GiN) and investigated their neurochemical composition. In addition, we used immunohistochemistry to evaluate the product of the immediate early gene, Egr-1, in control and 6-hydroxydopamine (6-OHDA)-lesioned rats to determine the regulation of PPN motor connections in the Parkinsonian state (Beckmann and Wilce 1997; Chaudhuri et al. 1997; O’Donovan et al. 1999).

## Methods

### Animals

All animal procedures used in this study were carried out under the authority of the Animals (Scientific Procedures) Act, 1986 (UK) and in accordance with the Society for Neuroscience policy of the use of animals in neuroscience research. Sprague-Dawley rats (200–300 g) were used for the tracer injections, 6-OHDA lesions and immunohistochemical analyses.

### 6-Hydroxydopamine lesions

Unilateral 6-OHDA lesions were carried out under anesthesia which was induced and maintained with isoflurane (Iso, Schering-Plough Ltd., Welwyn Garden City, UK). The animals were placed in a stereotaxic frame (David Kopf Instruments) and injected with desipramine (i.p.; 25 mg/kg in 0.9 % NaCl in dH_2_O; Sigma) 20 min before the 6-OHDA injections. A small craniotomy was made in the right hemisphere and 3 μl of a 6-OHDA solution (3 mg/ml of 6-OHDA hydrochloride salt; dissolved immediately before use in an ice-cold solution of 0.9 % NaCl and 0.02 % ascorbic acid in dH_2_O; Sigma) was injected (10 μl syringe; Hamilton 701RN; HA-763501; Jaytee Biosciences Ltd., Herne Bay, Kent, UK) into the medial forebrain bundle at the following co-ordinates: 4.5 mm posterior to Bregma, 1.3 mm lateral to Bregma and 7.9 mm ventral to the dura. Injections were carried out with the aid of a micropump at a rate of 0.5 μl/min. The syringe was left in place for 5 min before and after the injections and then slowly withdrawn.

The extent of the 6-OHDA lesion was assessed 14–15 days after the injection by challenge with a subcutaneous injection of apomorphine (0.05 mg/kg; 0.9 % NaCl, Sigma). A lesion was considered successful in animals that made 80 or more contraversive rotations in 20 min in response to the apomorphine injection. Only animals with successful lesions were subsequently used for this study. Typically 1 day after the apomorphine test, animals were injected with retrograde tracers into the STN and GiN.

### Tracer injections

Fluorescently labeled red and green retrobeads (red retrobeads: 530 nm excitation and 590 nm emission wavelengths; green retrobeads IX; 460 nm excitation and 505 nm emission wavelengths; Lumafluor, Inc, Durham NC, USA) were injected into the STN or GiN, respectively, of control and 6-OHDA-lesioned animals. Anesthesia was induced using 4 % isoflurane in O_2_ and maintained during surgery using an average of 2 % isoflurane (Iso, Schering-Plough Ltd., Welwyn Garden City, UK). The animals were placed in a stereotaxic frame (David Kopf Instruments, Tujunga, CA, USA), a small craniotomy was made above the STN (3.7 mm posterior to Bregma, 2.6 mm lateral to Bregma and 7.6 mm ventral to the dura) and 100 nl of red retrobeads were injected in the STN (1 μl syringe, 70 mm long; SGE, World Precision Instruments, Stevenage, UK). After withdrawal of the syringe, a second small craniotomy was made above the GiN (10.5 mm posterior to Bregma, 0.9 mm lateral to Bregma and 9.5 mm ventral to the dura), into which 150 nl of green retrobeads were injected. The syringe was left in place for 5 min before and after the injections and then withdrawn.

After 2 weeks of tracer injections, the animals were perfuse-fixed at the beginning of the dark phase of the 12:12 h light/dark cycle. They were deeply anesthetized using a mixture of ketamine (30 mg/kg, i.p.; Ketaset, Willow Francis, Crawley, UK) and xylazine (3 mg/kg, i.p.; Rompun, Bayer, Germany) and intracardially perfused with 0.1 M phosphate-buffered saline (PBS) followed by 4 % paraformaldehyde (PFA) in 0.1 M phosphate buffer (PB; pH 7.4) as fixative. Brains were removed and post-fixed in the same fixative for 0.5–1.5 h at room temperature. After washing, sagittal sections (50 μm thick) of the brainstem were then cut using a vibratome (Leica Microsystems, UK), collected in six series and stored in PBS containing 0.05 % sodium azide at 4 °C.

### Processing of the tissue and immunohistochemistry

Sections lateral and medial to the PPN (containing the STN and the GiN, respectively), were collected to localize the sites of the tracer injections under the fluorescent microscope. Two series of control sections were double-immunolabeled for calretinin and ChAT or calbindin and ChAT (one series of sections for each). They were washed three times in PBS, blocked for 1 h in normal donkey serum (NDS; Jackson Immunoresearch Laboratories Inc., West Grove, PA; 10 % in 0.3 % Triton X-100 in PBS) before primary antibodies against ChAT together with antibodies against calretinin or calbindin were added (ChAT raised in goat, AB144P, Millipore, Temecula, CA, 1:500 dilution; calbindin raised in mouse, CB300, Swant, Switzerland, 1:5,000 dilution; calretinin raised in rabbit, 7699/3H, Swant, Switzerland, 1:5,000 dilution, in 1 % NDS, 0.3 % Triton X-100 in PBS) and incubated overnight at 4 °C. They were then washed in PBS and the secondary antibodies were added in NDS: donkey anti-goat-AMCA, 1:100 dilution, and either donkey anti-mouse-Cy5 1:250 dilution or donkey anti-rabbit-Cy5 (Jackson Immunoresearch Laboratories Inc) 1:250 dilution in 1 % NDS, 0.3 % Triton X-100 in PBS and incubated at 4 °C overnight.

Preliminary incubations in control and 6-OHDA-lesioned animals were used to determine the most suitable transcription factor to detect changes in the PPN as a consequence of the lesion (data not shown). We incubated with antibodies raised against Fos B (sc-48, raised in goat, dilutions 1:250, 1:500; Santa Cruz Biotechnology, Inc., USA), Egr-1 (early growth response 1; sc-189, raised in rabbit, dilutions 1:2,000, 1:2,500; Santa Cruz Biotechnology, Inc) and phosphorylated cAMP response element-binding (pCREB; raised in rabbit, dilutions 1:50, 1:100, 1:200, 1:500; Cell Signaling Technology, USA). The main criteria for selection included detectable expression in the PPN and adequate signal-to-noise ratio to allow their quantification in retrogradely labeled neurons. Thus, whereas the Fos B expression was adequate, but showed low basal levels, the pCREB expression was abundant and compromised the accurate detection in the nuclei of neurons labeled with other markers. Only Egr-1 provided an optimal signal-to-noise ratio for the purpose of this study.

Sections from control and 6-OHDA-lesioned animals, with accurate tracer injections into the STN and GiN, were immunolabeled to reveal Egr-1 and ChAT. One of the six series of brain sections collected were rinsed in PBS (3 times, 10 min each) and blocked for 1 h in NDS (10 % in PBS), before adding primary antibodies (anti-Egr-1, raised in rabbit, Santa Cruz, 1:2,500; anti-ChAT, raised in goat, Millipore, 1:500; in 1 % NDS in PBS, incubated overnight at 4 °C). The following day, sections were washed in PBS then incubated overnight at 4 °C in secondary antibodies (donkey anti-rabbit-Cy5, 1:250; donkey anti-goat-AMCA, 1:100; Jackson Immunoresearch Laboratories Inc; in 1 % NDS in PBS). They were then washed 3 times in PBS, mounted on to slides with Vectashield mounting medium (Vector Laboratories Inc.) and stored in the dark at 4 °C until examined. Control reactions for all the experiments were performed by omitting each of the primary antibodies in turn, which revealed a complete absence of fluorescence for the omitted antibody.

### Image acquisition

Immunofluorescent and retrogradely labeled neurons were analyzed by capturing images using a LSM-710 (CarlZeiss, Germany) confocal microscope (20 × 0.8 NA dry objective lens) with an ApoTome-structured illumination system and using Colibri LED fluorescence as illumination source, or an Axio Imager M2 microscope (Plan-Apochromat 20 ×/0.8 M27 dry objective; Carl Zeiss, Germany). Multi-channel stacks of images were taken in the *Z*-plane using a digital camera (Axiocam HRm or HD Cam) in combination with acquisition software Axiovision 4.8.1 (Carl Zeiss AG, Germany). Confocal microscope images were obtained using the ZEN software version 5.8 (Plan-Apochromat 40 ×/1.3 oil DIC M27 objective). The software’s default settings were used for filters: Ch1-T1 504, Ch2-T2 560 and Ch3-T3 650 and the laser wavelengths were 488 nm for green retrobeads, 543 nm for red retrobeads and 633 nm for Cy5. The brightness and contrast of the images were subsequently adjusted in Photoshop (Adobe Systems Inc., Mountain view, CA, USA).

### Analysis of retrogradely labeled PPN neurons

The quantification and rostro-caudal distribution of STN- and GiN-projecting neurons that were positive for calbindin and ChAT or calretinin and ChAT were analyzed using a method based on the subdivision of the PPN into equally spaced segments, as described previously (Mena-Segovia et al. 2009). Using the center of the SNr, concentric circles at 300 μm intervals were drawn outwards to cover the entire extent of the PPN; the first two segments covered the SNr, then up to ten segments covered the PPN up to its caudal extremity (from S1 to S10). The limits of the PPN were defined by the cholinergic (ChAT-positive) neurons, with segment S1 being the most rostral and closest to the SNr and segment S10 being the most caudal.

Immunofluorescent and retrogradely labeled neurons were analyzed by capturing images of the entire PPN. Multi-channel stacks of images were taken in the *Z*-plane, each separated by approximately 3 μm. For each *Z*-plane level, squares of tissue were captured in the *X* and *Y* axes, each overlapping slightly with adjacent squares, to create an image of the whole PPN. Following the same method, low magnification images were captured (using a 5 × 0.16 NA dry objective) to include the entire PPN as well as the SNr. Using the center of the SNr as a reference point, concentric circles (330 μm apart) were drawn to delimit segments of the PPN (see Martinez-Gonzalez et al. 2012; Mena-Segovia et al. 2009) to allow the distributions of labeled neurons to be analyzed using Image J software. The distances between concentric circles were modified from previous analyses (see Mena-Segovia et al. 2009; Martinez-Gonzalez et al. 2012) as non-dehydrated sections shrank 10–13 % less than dehydrated sections (measurements were done in each of the analyzed brains in the *Z*-plane from the top to the bottom of the section, using Stereo Investigator software). This was confirmed by measurements taken between the center of the SNr and the superior cerebellar peduncle (approximately 1.9 mm lateral to Bregma; using Stereo Investigator software). In dehydrated sections, this distance was 4,125 ± 43 μm (*n* = 6; Mena-Segovia et al. 2009) as opposed to 4,659.4 ± 16.8 μm in non-dehydrated sections (mean ± SEM; *n* = 3).

For the evaluation of Egr-1 labeling, the analyses were performed as before but with a Zeiss fluorescent microscope attached to a Hamamatsu digital video camera and Stereo Investigator software. *Z*-plane stacks of images were acquired at intervals of 4 μm and the use of low magnification images to define the segments of the PPN was not necessary. Stereo Investigator software was able to define these segments, using the high magnification (20×) images, once the center of the SNr and the boundaries of the PPN were specified.

### Statistics

All data are presented as mean ± SEM; Student’s *t* test, Rank sum Mann–Whitney and ANOVA tests were performed using Sigma Plot software version 12.0 as appropriate.

## Results

### Ascending and descending motor pathways originate in the PPN

**Fig. 1:**
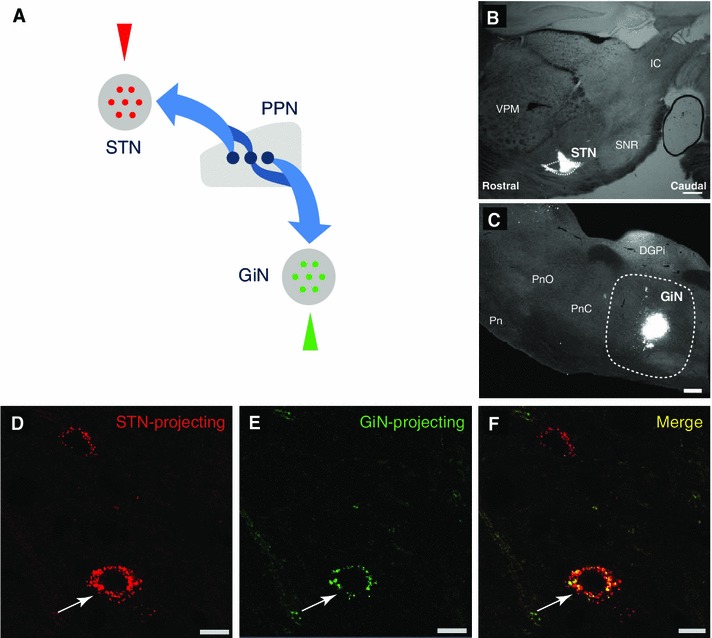
PPN neurons project to STN and GiN. **a** *Schematic* representation showing the sites of retrobead injections (*triangles*). Red retrobeads were injected in the STN and green retrobeads were injected in the GiN. **b**, **c** Light (**b**) and fluorescent (**c**) *micrographs* showing the injection sites for retrograde tracers in the STN (**b**) and GiN (**c**; *dotted lines* denote STN and GiN boundaries). **d**–f Fluorescent images showing a PPN neuron that projects to both the STN and GiN (*arrows*) as revealed by retrograde labeling (**d** and **e**, red and green retrobeads respectively; **f** *merged* *image*). *DPGi* dorsal paragigantocellular nucleus, *IC* inferior colliculus, *Pn* pontine nuclei, *PnO* nucleus pontis oralis, *PnC* nucleus pontis caudalis, *scp* superior cerebellar peduncle, *SNR* substantia nigra pars reticulata, *VPM* ventral posteromedial thalamic nucleus. *Scale bars* **b** and **c**: 500 μm; **d**–**f** 10 μm

Neurons of the PPN have extensive axonal projections. In some cases (e.g., cholinergic neurons), axon collaterals from individual neurons innervate both ascending (i.e., forebrain, basal ganglia) and descending (i.e., lower brainstem, medulla) targets (Mena-Segovia et al. 2008). In contrast, non-cholinergic neurons possess only one or two collaterals with exclusively ascending or descending projections (Mena-Segovia et al. 2008; Ros et al. 2010). In order to determine the extent of overlap of neurons with ascending and/or descending innervation to motor targets, we injected retrograde tracers into the STN (red retrobeads) and the GiN (green retrobeads) to label ascending and descending PPN projection neurons (Fig. 1a; *n* = 6). Injections in the STN covered ~80 % of the nucleus (Fig. 1b), and about 80 % of the tracer injection was confined to the border of the nucleus in each case that was subjected to detailed analysis. Injections in the GiN covered only a fraction of the structure (Fig. 1c) and in most cases the totality of the tracer injection was located within the GiN borders, as determined by the brain atlas (Paxinos and Watson 1986), with only traces in the injection track situated outside the border. We observed retrogradely labeled neurons with red (STN-projecting neurons; Fig. 1d) or green (GiN-projecting neurons; Fig. 1e) retrobeads in the PPN 10–15 days following the tracer injections. The borders of the PPN were defined by ChAT-expressing neurons as described previously (Mena-Segovia et al. 2009). Retrobeads in the PPN were localized predominantly in the soma and had a distinctive punctate appearance (Fig. 1d, e). A small proportion of both types of retrobeads were detected outside the soma, presumably corresponding to the processes of the retrogradely labeled neurons, but the punctate pattern of expression did not allow their characterization. Only cell bodies were considered for further analysis. The discrete localization of the retrograde markers allowed the unequivocal detection of double-labeled neurons (Fig. 1f).

**Fig. 2:**
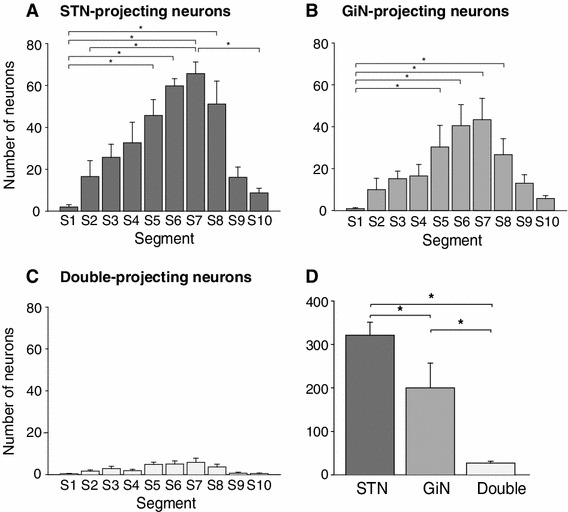
Two motor pathways originate from distinct neuronal subsets in the PPN. **a**–**c** *Histograms* showing the number of neurons per PPN segment that project to the STN (**a**), GiN (**b**), or both nuclei (**c**). The neuronal populations projecting to the STN and to the GiN show similar but heterogeneous distributions across the rostro-caudal axis of the PPN. The greatest numbers of projection neurons per segment were detected in S5–S8. In contrast to the single-projection neurons, the number of double-projection neurons was considerably smaller and did not show a significantly heterogeneous rostro-caudal distribution. **d** *Histogram* showing the total number of STN-, GiN- or double-projection PPN neurons. The number of double-projecting PPN neurons was significantly smaller than the number of neurons projecting to either of the targets alone. For each panel, *columns* represent means, *error bars* indicate SEM and *asterisks* denote significant differences between groups or segments (*p* < 0.05; *n* = 6)

We then analyzed the number and distribution of STN- and GiN-projecting neurons across the rostro-caudal extent of the PPN (Mena-Segovia et al. 2009; Martinez-Gonzalez et al. 2012). We observed that neurons retrogradely labeled only from the STN (STN-only; Fig. 2a) and those only labeled from the GiN (GiN-only; Fig. 2b) have a similar, non-uniform rostro-caudal distribution across the PPN, with their largest numbers in the caudal half of the nucleus (ANOVA on ranks, Kruskal–Wallis (K–W); STN: H_9_ = 41.98; GiN: H_9_ = 33.25; *p* < 0.001; *n* = 6). Statistical differences were mostly observed between segments 5–8 and the most rostral and caudal segments (Dunn’s test; *p* < 0.05; see Fig. 2a, b for specific comparisons). A much lower number of neurons retrogradely labeled from both structures (double-projecting neurons) were detected but they were also non-uniformly distributed across the PPN (Fig. 2c; ANOVA on ranks K–W, H_9_ = 25.99; *p* < 0.002; no differences were observed between specific PPN segments, Dunn’s test; *p* > 0.05). The mean total number of neurons retrogradely labeled from the STN only (321 ± 30) was somewhat larger that the number detected projecting only to the GiN (200 ± 40). These differences may relate to several factors including the site of the deposits of retrograde tracers, the proportion of the target structure occupied by the tracer deposits, the size/density of the axonal fields in the STN and GiN and the activity of the projection neurons. It is thus inappropriate to make comparisons between them, suffice to say that similar orders of magnitudes of neurons project to both targets. The numbers of neurons retrogradely labeled from both structures was much smaller (27 ± 4). The factors listed above, as well as topography of the projections may lead to false-negatives and small numbers of double-labeled neurons. However, the consistently small number of double-labeled neurons suggests that numbers are in fact low, or there are differences in the size and/or density of collaterals in different structures that could lead to false negatives. These data show that the ascending and descending PPN projections to motor structures largely originate from distinct subsets of neurons.

### Neurochemical heterogeneity of the motor output of PPN neurons

**Fig. 3:**
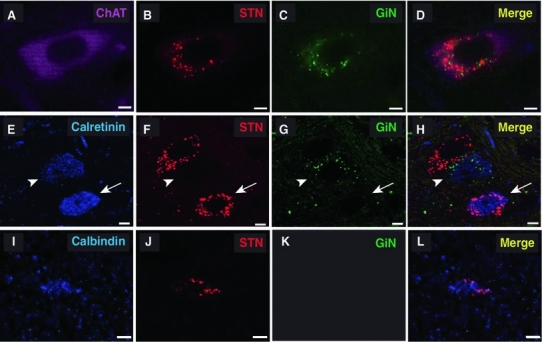
Neurochemical heterogeneity of PPN neurons giving rise to motor pathways. **a**–**d** Fluorescent images showing a cholinergic PPN neuron (ChAT positive **a**) that projects to both the STN (**b**) and GiN (**c** *merged image* in **d**). **e**–**g** Fluorescent images of two calretinin-positive PPN neurons (**e**), one projecting to the STN (**f** *arrow*) and the other projecting to the GiN (**g** *arrowhead*; *merged image* in **h**). **i**–**l** Fluorescent images of a calbindin-positive PPN neuron (**i**) projecting to the STN (**j**; *merged image* in **l**), but not to the GiN (**k**). *Scale bars* 5 μm

The PPN contains subclasses of neurons that express distinct neurochemical markers. While cholinergic neurons define the borders of the PPN itself, they account for only about 20 % of all neurons in the PPN (Wang and Morales 2009; Mena-Segovia et al. 2009). Recently, we showed that calbindin and calretinin are commonly expressed in PPN neurons and their numbers are similar in magnitude to those of the cholinergic neurons. As these calcium-binding proteins are also expressed in GABAergic and glutamatergic neurons, we tested for the presence of ChAT (Fig. 3a–d), calretinin (Fig. 3e–h) or calbindin (Fig. 3i–l) immunoreactivity in retrogradely labeled neurons. In confirmation of previous work (Mena-Segovia et al. 2008), we observed cholinergic neurons retrogradely labeled from the STN and/or the GiN (Fig. 3a–d). Calretinin-positive neurons were observed to project to both targets (Fig. 3f, g), but immunopositive neurons projecting to both structures were not detected (Fig. 3h), suggesting that different populations of calretinin-expressing neurons project to the STN and the GiN. In contrast, calbindin-positive neurons were only observed to project to the STN (Fig. 3i–l; *n* = 6). These results suggest that the PPN neurons that innervate the STN and the GiN are neurochemically heterogeneous.

**Fig. 4:**
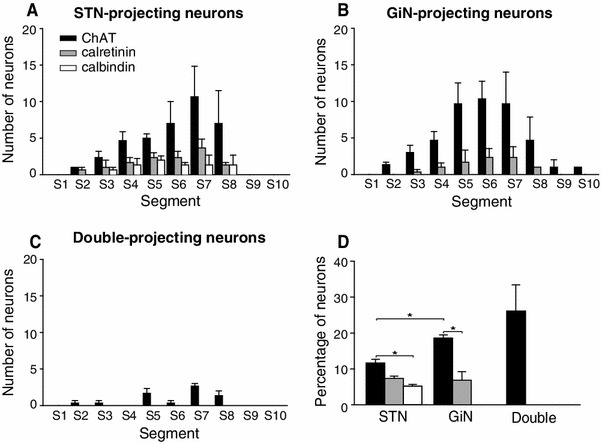
Ascending and descending motor pathways express distinct neuronal markers. **a**–**c** *Histograms* showing the numbers of STN- (**a**), GiN- (**b**) and double-projecting (**c**) PPN neurons that are cholinergic, calretinin-positive or calbindin-positive, per PPN segment. Of the single-projection neurons, only cholinergic neurons follow a similar pattern of rostro-caudal distribution (S5–S8) for both STN- and GiN-projecting neurons (see Fig. 2a, b). Calbindin immunoreactivity was not detected in PPN neurons projecting to the GiN. Also evident was the lack of calretinin and calbindin expression in double-projection neurons. **d** *Histogram* showing the percentages of STN-, GiN- and double-projecting PPN neurons that are cholinergic (ChAT-positive), calretinin-positive or calbindin-positive. Only cholinergic markers, out of the three makers tested, were detected in double-projection neurons. For each panel, *columns* represent means, *error bars* indicate SEM (*n* = 3)

Cholinergic neurons were the most commonly retrogradely labeled neurons and those projecting to the STN (Fig. 4a) or the GiN (Fig. 4b) showed similar, non-uniform distributions, and tended to concentrate in the caudal half of the PPN (STN: ANOVA on ranks K–W, H_9_ = 22.745, *p* = 0.007; GiN: 1-way ANOVA, F_8,18_ = 3.29, *p* = 0.017). However, some small differences were observed: whereas the numbers of STN-projecting, ChAT-positive neurons increase gradually in a rostro-caudal gradient peaking at segment 7, the majority of GiN-projecting, ChAT-positive neurons are concentrated in segments 5–7. The number of double-projecting ChAT-positive neurons was much smaller than the number of neurons projecting to STN-only or GiN-only, and did not show any trend in their rostro-caudal distribution, even though they were non-uniformly distributed (Fig. 4c; 1-way ANOVA, F_8,18_ = 6.11, *p* < 0.001). STN- and GiN-projecting, calcium-binding protein-positive neurons were smaller in number and did not show a non-uniform distribution (Fig. 4a, b). The number of STN-only projecting neurons that express ChAT was significantly larger than those expressing calbindin (*t*_4_ = 3.26, *p* = 0.031). In addition, the number of GiN-only projecting neurons that express ChAT were significantly larger (by a factor of two at least) than the number of calretinin-positive neurons projecting to the same target (*t*_4_ = 3.59, *p* = 0.023). Furthermore, a significantly larger number of ChAT-positive neurons were retrogradely labeled from the GiN than the STN (*t*_4_ = −5.026, *p* = 0.007). Finally, about a third of the double-projection neurons were cholinergic (Fig. 4d). These results support the notion of a differential innervation of PPN targets arising from neurochemically distinct subpopulations of PPN neurons.

### Differential regulation of PPN-projecting neurons in Parkinsonism

**Fig. 5:**
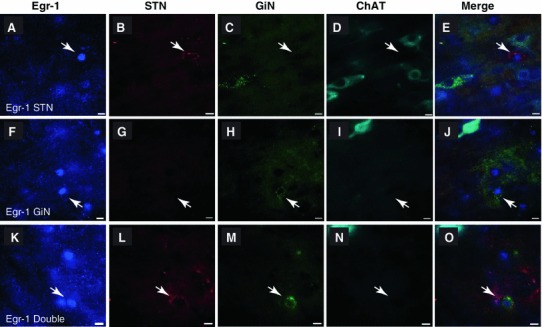
Immediate early gene expression in the Parkinsonian rat. **a**–**e** Fluorescent images showing an Egr-1-positive PPN neuron (**a**, *arrow*) that was retrogradely labeled from the STN (**b**), but not the GiN (**c**), and was non-cholinergic (**d**; *merged image* in **e**). **f**–**j** Fluorescent images of an Egr-1-positive PPN neuron (**f**, *arrow*) that was not retrogradely labeled from the STN (**g**), but was labeled from the GiN (**h**; **i**, also non-cholinergic; merged image in **j**). K–O Fluorescent images of an Egr-1-positive PPN neuron (**k** *arrow*) that projects to both the STN (**l**) and GiN (**m**; **n**, also non-cholinergic; merged image in **o**). *Scale bars* 10 μm

**Fig. 6:**
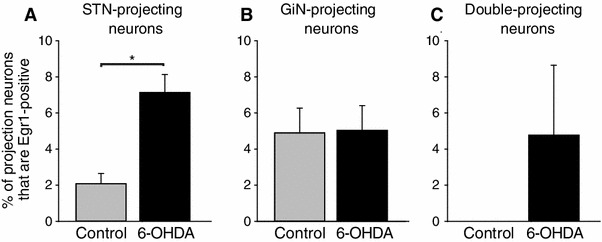
Differential regulation of motor pathways in the PPN of Parkinsonian rats. **a**–**c** *Histograms* showing the percentages of STN- (**a**), GiN- (**b**) and double-projection (**c**) PPN neurons that are Egr-1-positive in control and 6-OHDA lesioned animals. For each panel, *columns* represent means, *error bars* indicate SEM and the *asterisk* denotes a significant difference between the control and 6-OHDA lesioned groups for STN-projecting neurons (*p* = 0.006; control *n* = 5; 6-OHDA *n* = 8)

The impaired activity of the PPN in PD has been associated with some of the symptoms of the disease but the nature of the neurons involved in these changes is not yet understood. We therefore tested the basal levels of activity (i.e. no stimulation and immediately before the activity period) as indicated by the expression of the immediate early gene (IEG) Erg-1, in the two motor pathways in controls and in the 6-OHDA rat model of PD. Two–four weeks following unilateral dopamine depletion, we injected retrobeads into the STN and GiN. After a further 10–15 days survival, to allow transport of the tracers, they were perfuse-fixed at the beginning of the activity period (dark phase of the cycle) and processed to reveal Egr-1 immunoreactivity in retrogradely labeled PPN neurons (Fig. 5). Egr-1 immunolabeling was localized in the nuclei of neurons (Fig. 5a, f, k). Egr-1 expression was present in the PPN of control (230 ± 86, *n* = 5) and 6-OHDA animals (315 ± 62, *n* = 8; no statistical difference), and in a proportion of STN-only projecting neurons (Fig. 5a–e), GiN-only projecting neurons (Fig. 5f–j) and double-projection neurons (Fig. 5k–o). Egr-1 expression was not detected in the nuclei of cholinergic neurons (Fig. 5d, i, n). In STN-projecting neurons, there was a significant difference in the proportion expressing Egr-1 between control and 6-OHDA rats (Fig. 6a; *t*_9_ = 3.599, *p* = 0.006; control *n* = 5, 6-OHDA *n* = 8). Thus, although low in absolute numbers, Parkinsonian rats showed a three-fold increase in the number of STN-only projecting neurons that are also immunopositive for Egr-1. In contrast, no difference was observed between control and 6-OHDA rats in the neurons that were retrogradely labeled from the GiN (Fig. 6b). Furthermore, we did not detect any double-projection neuron expressing Egr-1 in control animals. This contrasted with the 6-OHDA group that showed consistent Egr-1 expression in double-projection neurons across all animals (Fig. 6c). These data suggest that ascending and descending pathways from the PPN are differentially regulated in the Parkinsonian rat.

## Discussion

We present here evidence of two distinct, largely non-overlapping pathways arising from the PPN that innervate different motor structures involved in different functions. We further show that these pathways are neurochemically distinct and are differentially affected in the Parkinsonian rat. Thus, PPN neurons projecting to the STN are heterogeneous, showing expression of ChAT, calbindin and calretinin, and an increased level of activity following a 6-OHDA lesion of the SNc, as suggested by the expression of Egr-1. In contrast, PPN neurons projecting to the GiN are less heterogeneous, they do not express calbindin and a larger proportion is cholinergic, but they do not show changes in the expression of Egr-1, following a 6-OHDA lesion. Our results provide a basis for understanding distinct motor pathways of the PPN and support a role of PPN neurons in the upstream changes in the basal ganglia following dopamine depletion.

### The motor function of the PPN

The PPN has been traditionally considered as a part of the MLR. Early experiments showed that electrical stimulation in the region of the PPN was able to elicit locomotion (Skinner et al. 1990). The results of more recent studies using more refined techniques suggest that PPN maintains a close relationship with the MLR, and its downstream projections inhibit locomotion. Thus, through its descending projections, mediated in part by the reticulospinal neurons of the GiN, the effect of PPN activation would be the excitation of inhibitory interneurons in the spinal cord and the modulation of the excitatory output of the MLR (Takakusaki 2008; Takakusaki et al. 2004). On the other hand, the PPN is known to have close interconnectivity with many elements of the basal ganglia (Mena-Segovia et al. 2004), including the STN (Nomura et al. 1980; Edley and Graybiel 1983; Hammond et al. 1983). Activation of the STN, driven at least in part by direct cortical inputs, increases the inhibition of basal ganglia targets, mediated by its connections with the substantia nigra pars reticulata (SNr). Thus, an increased STN output would lead to an increased inhibition of the reticular targets of the SNr. In vitro experiments have shown that the overall effect of PPN activation on STN is excitation (Hammond et al. 1983). Hence, the net effect of the ascending (activation of the STN and basal ganglia output) and descending (activation of inhibitory spinal neurons) output of the PPN is likely to be motor inhibition.

Based on the above rationale, and the position of the PPN in the inhibitory output stream of the basal ganglia (i.e., SNr), a dysfunction in the PPN output is likely to aggravate the motor impairment in PD. Animal models of PD have shown some degree of change in the activity of the PPN (Nandi et al. 2002; Breit et al. 2001; Aravamuthan et al. 2008), but the nature and causes for such changes remain elusive. The neurochemical, electrophysiological, topographical and functional heterogeneity of PPN neurons (Mena-Segovia et al. 2008, 2009; Ros et al. 2010; Martinez-Gonzalez et al. 2012; Wang and Morales 2009; Boucetta and Jones 2009; Alderson et al. 2008) suggests it is impractical to attempt to understand the output of the PPN based simply on activation or inhibition. In the present paper, we endeavored to take into consideration the heterogeneity by incorporating different variables (projection targets, topography, neurochemical makers and level of activity). Our findings thus support the hypothesis that activity of different subclasses of neurons in the PPN is differentially regulated in Parkinsonian animals.

### Functionally distinct motor pathways in the PPN

Our results show that a subpopulation of PPN neurons that have ascending projections to the STN are distinct from the neurons that have descending projections to the GiN (Mena-Segovia et al. 2008; Ros et al. 2010). We did not observe a distinct topographical gradient between these projections. This finding concurs with a previous report on the distribution of STN-projecting neurons (Kita and Kita 2011) and suggests that the PPN projects largely separately to these two motor structures but the projections arise from neurons that are intermingled in the same regions of the PPN. We have previously identified local synaptic contacts arising from different subclasses of cholinergic and non-cholinergic neurons within the PPN (Mena-Segovia et al. 2008; Ros et al. 2010). It is thus possible that the axon collaterals of the projection neurons in one pathway contact the projection neurons from the other pathway and vice versa. When considering the heterogeneity of afferents to the PPN that arise from diverse neuronal systems (reviewed in Martinez-Gonzalez et al. 2011), this suggests an integrative role within PPN microcircuits. In turn, the distribution of cholinergic, GABAergic and glutamatergic neurons (Mena-Segovia et al. 2009; Wang and Morales 2009; Martinez-Gonzalez et al. 2012) suggests that the rostral PPN is predominantly inhibitory (GABAergic) whereas the caudal PPN is predominantly excitatory (glutamatergic). Thus, it is likely that both motor projections (i.e., STN and GiN) are predominantly glutamatergic, and the fact that they contain a different balance of calcium-binding proteins suggests that different subtypes of glutamatergic neurons may be involved. Nevertheless, it should be noted that a GABAergic component is also present (Bevan and Bolam 1995).

To identify the neurochemical nature of the STN- and GiN-projecting neurons, we used immunohistochemistry for ChAT, calbindin and calretinin. The two calcium-binding proteins have been shown by in situ hybridization to be expressed in a large number of GABAergic and glutamatergic neurons of the PPN (Martinez-Gonzalez et al. 2012). We observed that the number of cholinergic neurons projecting to either target was greater in the caudal PPN compared to its rostral portion, suggesting that cholinergic-mediated excitation of motor structures arises largely in the caudal PPN. Interestingly, we detected more cholinergic neurons retrogradely labeled from the GiN than the STN despite the fact that the tracer deposits included a much smaller proportion of the GiN than the STN. We also detected ChAT expression in neurons projecting to both structures, which is consistent with the collateralization reported in individually labeled and reconstructed neurons (Mena-Segovia et al. 2008). About two-thirds of the double-projection neurons were identified as non-cholinergic. In contrast to the cholinergic neurons, calretinin-positive neurons were observed to project to STN and GiN, but never in the same neurons, whereas calbindin-positive neurons were only found to project to the STN. Our findings thus suggest that the output of the PPN to the STN and GiN is highly complex and heterogeneous. Given the caveats of false-negatives in retrograde labeling studies (see above), our findings suggest that sub-populations of cholinergic neurons project to either the STN or the GiN and a sub-population projects to both structures. Similarly, sub-populations of non-cholinergic neurons (presumably GABAergic and glutamatergic) project to either the STN or the GiN and sub-populations project to both structures (not previously identified). The heterogeneity is even more complex, not just because of their potentially GABAergic or glutamatergic nature, but also as consequence of the differential expression of the calcium-binding proteins. Our findings are largely consistent with our previous in vivo electrophysiological characterization of cholinergic and non-cholinergic neurons in the PPN (Mena-Segovia et al. 2008; Ros et al. 2010), although we did not detect non-cholinergic neurons protecting to both structures. Non-cholinergic neurons with descending projections were shown to have distinct electrophysiological properties to non-cholinergic neurons with ascending projections, supporting the notion of functional differences between these two pathways in normal animals (Ros et al. 2010).

### Immediate early gene expression in control and 6-OHDA lesioned rats

The analysis of the two pathways following a 6-OHDA lesion of dopamine neurons in the SNc, showed an increase in the number of active STN-projecting neurons, as indicated by Egr-1 up-regulation. This was also most evident in double-projection neurons, suggesting that overall, PPN neurons with ascending projections (both STN-only and double-projection) show Egr-1 up-regulation. Because Egr-1 expression has been extensively associated with increased neuronal activity (for reviews see Beckmann and Wilce 1997; O’Donovan et al. 1999), we interpret these findings as an increased activation of PPN neurons with ascending projections, and consequently, an increased synaptic drive of their target structures. Although our data show no Egr-1 expression in cholinergic neurons, suggesting that they may not be affected in this animal model, we cannot rule out the existence of false negatives. The difference in the expression between the ascending and descending pathways may reflect a selective excitatory drive of STN-projecting neurons and a potential increased inhibition of GiN-projecting neurons (not detectable by the basal level of IEGs expression), in agreement with the data from other groups proposing an increased excitation (Breit et al. 2001; Orieux et al. 2000; Barroso-Chinea et al. 2011) or an increased inhibition (Nandi et al. 2002) in the Parkinsonian PPN. In this way, an imbalanced output between the two motor pathways may underlie the gait disturbances associated with PD.

In conclusion, our data provide evidence of functional differences associated with distinct neuronal subtypes in the PPN that contribute to different neuronal circuits. We and others have proposed a functional topography in the PPN associated with the afferent and efferent connectivity. Here, we show evidence of functional divergence among intermingled neurons located in the same functional domains of the PPN. Further detailed characterization of the neuronal circuits that constitute the PPN will be necessary to understand its role in behavior and its contribution to pathological processes that distinguish some neurological disorders.
